# The influence of circadian rhythm disruption during Ramadan on metabolic responses to physical activity: a pilot study

**DOI:** 10.3389/fnins.2025.1542016

**Published:** 2025-02-24

**Authors:** Najeha Rizwana Anwardeen, Khaled Naja, Shamma Almuraikhy, Maha Sellami, Hadaia Saleh Al-Amri, Nebu Philip, Faleh Tamimi, Ahmed Agil, Mohamed A. Elrayess

**Affiliations:** ^1^Biomedical Research Center, QU Health, Qatar University, Doha, Qatar; ^2^College of Sport Sciences, Sport Coaching Department, Qatar University, Doha, Qatar; ^3^College of Dental Medicine, QU Health, Qatar University, Doha, Qatar; ^4^Department of Pharmacology, BioHealth Institute Granada (IBs Granada), Neuroscience Institute (CIBM), School of Medicine, University of Granada, Granada, Spain; ^5^College of Medicine, QU Health, Qatar University, Doha, Qatar

**Keywords:** circadian rhythm, exercise, intermittent fasting, metabolomics, metabolism

## Abstract

**Background:**

Circadian rhythms and sleep patterns are important regulators of metabolic health. During Ramadan intermittent fasting (RIF), the sleep–wake cycles are often disrupted, which can affect physical activity (PA) and related metabolic responses. Limited knowledge is available on how sleep disruption influences PA in the general population during RIF. This pilot study aimed to examine the metabolic responses to moderate PA under normal and disrupted sleep patterns during RIF.

**Methods:**

A pilot study was conducted on 12 participants comprising of individuals with normal (*n* = 5) and disrupted sleep patterns (*n* = 7). Blood samples were collected, and measurements of clinical traits, cytokines, homeostasis model assessment of insulin resistance (HOMA-IR) and metabolic profiles were performed before and after intervention. Orthogonal partial least square – discriminant analysis (OPLS-DA) and linear regressions were performed to assess metabolic responses to PA during RIF under different patterns.

**Results and conclusion:**

Fasting participants with normal sleep patterns exhibited lower HOMA-IR (*β* = −0.416, *p* = 0.047) in response to PA compared to those with disrupted sleep. Additionally, they demonstrated more efficient lipid utilization during PA, characterized by reduced diacylglycerol levels, which could enhance insulin sensitivity and lower the risk of type 2 diabetes. In contrast, fasting participants with disrupted sleep patterns experienced metabolic stress, marked by significant depletion of polyunsaturated fatty acids (PUFAs), monounsaturated fatty acids (MUFAs), and plasmalogens in response to PA. These changes were associated with increased inflammation and oxidative stress, potentially leading to metabolic dysregulation.

## 1 Introduction

Human physiology varies throughout the 24-h cycle, encompassing changes at every stage, from the cellular (Cuesta et al., 2015) and metabolic (Chua et al., 2013) levels, to physiological and behavioral levels (Morris et al., 2015; Imeri and Opp, 2009). We have successfully evolved to synchronize metabolic activities to a 24-h day and sleep–wakefulness dependent systems and circadian rhythm (CR) are in charge of this 24-h variance. Sleep–wake systems and CR are essential for energy metabolism (Rynders et al., 2020; Zitting et al., 2018) and associated habits such as eating and exercise (Arble et al., 2015; Simon et al., 1998). For instance, growth hormone levels in the blood are mostly reliant on sleep, with the majority of secretion occurring during slow-wave sleep (Czeisler and Klerman, 1999). In contrast, cortisol levels peak in the early morning hours and are mostly regulated by the circadian cycle (Czeisler and Klerman, 1999). Similarly, energy expenditure peaks during waking hours and falls during sleeping hours (Jung et al., 2011).

Interestingly, the capacity of a human to predict and optimize metabolic processes is compromised if the circadian rhythms are disturbed or misaligned (Arble, 2023). The term “circadian misalignment” refers to a number of circumstances, including when sleep–wakefulness, and fasting-food intake cycles are not in sync with internal circadian time or external time, or when central and peripheral rhythms are not concurred (Chaput et al., 2023). Disturbances in the circadian rhythm have been associated with a number of cardiometabolic conditions, such as decreased insulin sensitivity, elevated markers of systemic inflammation, elevated blood pressure, decreased energy expenditure, and obesity (BaHammam and Almeneessier, 2020). Ramadan intermittent fasting (RIF) is an example of a pattern that can potentially disrupt the circadian rhythm and sleep–wake cycle as it involves a distinct pattern of dry and diurnal fasting for 29–30 days straight from sunrise to sunset followed by food-intake during the night hours (Patterson and Sears, 2017; Obaideen et al., 2022). Current literature has highlighted numerous advantageous metabolic, inflammatory, and anthropometric effects such as the improvement of body mass index (Jahrami et al., 2020), metabolic syndrome symptoms (Faris et al., 2020), inflammatory and stress markers (Faris et al., 2019), glucose metabolism (Faris et al., 2020), and promote longevity and healthier aging (Almuraikhy et al., 2024) during RIF.

Although, RIF has no direct effect on the circadian rhythm provided if the meals are strictly limited to the early evening and predawn hours and there is enough sleep during the night (BaHammam and Almeneessier, 2020). However, most individuals no longer follow the normal exposure to the natural sleep–wake cycle. People dine late into the night, remain active and exposed to light long after sunset, and wake up to alarm clocks hours later than they would ordinarily wake up. Moreover, there is evidence of reduced habitual physical activity of individuals during the fasting period (Farooq et al., 2021). Ramadan is typically regarded as a time of relative physical inactivity, increasing the risk of obesity and insulin resistance (AlMuraikhy et al., 2022). Both physical activity (PA) and IF have been postulated to have beneficial effects on health (Lessan et al., 2018). PA during fasting state is associated with increased fat metabolism compared to after food intake period, thereby emphasizing on the positive health outcomes of exercise performed when fasting (Lipert et al., 2021).

Notably, fundamental components of health are sleep and activity, and metabolic health is stressed by both circadian misalignment and insufficient sleep, which are linked to negative health consequences such as an elevated risk of obesity, cardiovascular disease, hypertension, dyslipidaemia and type 2 diabetes (Chaput et al., 2023; Chaput et al., 2020). While a recent study found that sleep disturbances during Ramadan negatively affect the physical performance of professional athletes (Lipert et al., 2021), there remains a significant knowledge gap regarding how disrupted sleep influences physical activity responses in the general population. To the best of our knowledge, no studies have addressed this issue outside of elite athletic contexts, and so the broader metabolic implications in non-athletic individuals are largely unexplored. We hypothesized that combining RIF and PA may increase the health advantages of fasting people, provided they adhere to their normal sleeping schedules. Sleep disturbances may attenuate these benefits by changing the metabolic pathways, such as disrupting lipid homeostasis, and promoting inflammation. This study aimed to assess the metabolic response to physical activity during RIF under both normal and disrupted sleep conditions which will provide novel insights into the role of circadian alignment in optimizing exercise-related metabolic responses. Given the observed reduction in daily physical activity during Ramadan (Farooq et al., 2021), particularly during fasting hours, our study aimed to assess the metabolic response to physical activity introduced during RIF under normal and disrupted sleep pattern in general population. By addressing this gap, our findings contribute to a growing field on the importance of sleep and circadian regulation in metabolic health and emphasize the need for lifestyle recommendations during Ramadan to maximize the benefits of fasting and exercise.

## 2 Methods

This study was conducted as a pilot study to investigate the metabolic responses to moderate physical activity in individuals with normal versus disrupted CR. The primary objective was to explore the preliminary changes that could be linked to variations in circadian rhythm and to analyze the study design’s viability for a more extensive investigation.

### 2.1 Participants

Twelve female students from Qatar University were enrolled during the month of Ramadan. These females (aged 20–30) volunteered to take part in the study. All participants provided consent form prior to participation. All protocols were approved by Qatar University (QU-IRB 1798-EA/23) as per regulations of Qatar Ministry of Public Health (MoPH). The inclusion criteria were as follows: BMI between 20 and 30 kg/m^2^, be free of cardiovascular disease, glaucoma, blood clots, muscle degeneration, cancer, and other autoimmune illnesses such as T2D or cancer. The disruption in circadian rhythm in our cohort was due to the habitual changes associated with Ramadan fasting.

In this study, participants self-reported their habitual sleep–wake cycles. Based on these reports, they were divided into two groups: those who maintained a normal circadian rhythm by sleeping during the night (*n* = 5) and those who exhibited a disrupted rhythm by sleeping during the day and remaining awake at night (*n* = 7). Groups were formed without intentional selection where age, health, and lifestyle were equally distributed by chance, allowing for fair comparison between the two groups. Clinical measurements and blood samples were taken before and after 4 weeks training period during Ramadan month.

### 2.2 Physical activity

A moderate intensity exercise (MIE) regimen was followed by all qualified individuals for three times per week during the afternoon (fasting hours) from 1 pm to 3 pm. Based on the American Society of American College of Sports Medicine (ACSM)’s recommendations (Haskell et al., 2007), training sessions included moderate (13–15 Borg scale, 50–70% VO_2_max) aerobic workouts (walking and jogging) which lasted for 30 min at a time. The intensity of the activities was determined by individual’s muscle and cardiorespiratory fitness. The six-minute walking test distance (6WT) is a submaximal assessment utilized to quantify the distance a participant can traverse on a flat surface within a six-minute duration. The Metabolic Equivalent of Task (MET) values were adjusted based on IPAQ responses to quantify daily activities. MET was utilized for intensity and energy expenditure, expressed similarly for individuals of different weights. The exercise intensity was evaluated using Borg scale test using the Borg rating of perceived exertion (RPE) (Borg, 1990). It was assessed for each participant following the 6WT and the training session. A person’s perception of their effort and exertion, dyspnoea, and exhaustion during an exercise can be measured with the Borg scale.

### 2.3 Metabolomics and statistical analysis

All participants’ serum samples were subjected to untargeted metabolomics utilizing Metabolon’s platform in accordance with established guidelines (Al-Khelaifi et al., 2018). The metabolomics data consisting of 259 unknown identities and 1,039 known identities were median scaled. The minimum value across batches from the median-scaled data was used to impute missing values before natural log transformation. The unknown metabolites were removed from further statistical analysis. To evaluate the data’s quality, principal component analysis (PCA) was used. Orthogonal partial least square-discriminant analysis (OPLS-DA) model was used to identify the greatest discriminant metabolites associated with physical activity under normal and disrupted CRs separately.

Univariate analyses were conducted in two stages: comparative analysis and interaction effects. The former included paired t-test between pre- and post- physical activity performed separately for normal and disrupted CRs to compare the change in the metabolite levels in different CR conditions. The latter analysis used a mixed-effects model to assess the interaction between CR and activity to determine if the effect of physical activity on metabolic profile differs by sleep pattern. The *p*-values were adjusted using false discovery rate (FDR) correction for both analyses. Additionally, functional enrichment analysis was performed on all nominally significant metabolites list from the comparative analysis using Wilcoxon sum of ranks and p-values were adjusted by the FDR correction. This was performed to investigate the over-represented sub-pathways using the metabolites in different CR conditions. The sub-pathways were previously predefined using Metabolon, and those with less than three top hits were dropped. Spearman’s correlation was performed between clinical parameters and relevant metabolites.

## 3 Results

### 3.1 Summary of participant clinical profile

Although none of the characteristics exhibited statistically significant variations between the two groups, HOMA-IR, LDL, TNF-*α*, total cholesterol, and triglycerides showed a decrease in normal group and an increase in the disrupted group (Table 1). Supplementary Table S3 shows the baseline comparison between the normal CR and disrupted CR groups.

**Table 1:** General characteristics of participants categorized by circadian rhythm (normal vs. disrupted) and training (before vs. after).

	Normal circadian rhythm (*n* = 5)		Disrupted circadian rhythm (*n* = 7)	
Age	21 (20–22)		22 (22–25.5)	
Height m	1.61 (1.6–1.62)		1.59 (1.56–1.64)	

	Before training	After training	*p*-value	Before training	After training	*p*-value
BMI	25.8 (21.7–34.1)	25.2 (21.8–34.3)	0.349	23.4 (22–26.15)	23 (22.05–26.15)	0.351
MET	1,686 (591–2,868)	1,230 (1155–1788)	1	1,097 (983–2034)	1873 (1298.25–2,178)	0.447
6WT Distance (m)	600 (513–630)	790 (660–800)	0.178	600 (544.5–612)	630 (625.5–678)	0.15
Handgrip (L)	19 (18.6–19)	21.4 (20–21.8)	0.343	24.1 (19.55–25.9)	25.9 (21.85–28.6)	0.040
Handgrip (R)	22.7 (18.2–24)	22.1 (21.1–24)	0.584	25.2 (19.15–26.8)	25.2 (24.1–27.4)	0.057
Insulin (mU/L)	16.77 (10.42–16.77)	18.75 (12.92–19.17)	0.418	8.54 (8.38–16.92)	10.42 (9.27–14.27)	0.933
FBS (mmol/L)	5.3 (4.8–5.3)	5.2 (5.2–5.4)	0.279	5.3 (4.9–5.45)	5.4 (5.25–5.6)	0.248
HOMA IR	3.93 (3.9–3.97)	2.21 (2.2–4.35)	0.281	2.09 (1.96–3.83)	2.47 (2.19–3.67)	0.8
Total Cholesterol (g/dl)	178 (159–191)	165 (158–172)	0.418	164 (153.5–166.5)	173 (160–189)	0.151
Triglycerides (g/dl)	66 (62–67)	58 (38–88)	0.584	55 (48–60)	59 (44.5–74)	0.673
HDL (g/dl)	48 (40–52)	46 (45–53)	1	54.5 (50–67.25)	55 (44–58.5)	0.787
LDL (g/dl)	111 (94–120)	110 (93–111)	0.892	95 (77–103.5)	102 (82.5–133.5)	0.108
HbA1C	5.15 (5.02–5.28)	5.09 (5.01–5.16)	1	5.1 (4.75–5.3)	5.28 (5.14–5.42)	0.295
IL 6 (pg/ml)	18.2 (8.82–24.77)	24.77 (24.77–24.77)	1	30.68 (24.77–39.38)	24.77 (24.77–24.77)	1
TNF alpha (pg/ml)	2.19 (0.43–5.76)	0.43 (0.43–0.43)	0.371	0.43 (0.43–3.07)	2.19 (0.43–3.95)	1

Data are presented as median (IQR) for all continous variables after Shapiro–Wilks normality test. Pre-/post-training period are compared using paired Wilcoxon sum of ranks test.

### 3.2 Global metabolic signature of physical activity in individuals with normal and disrupted circadian rhythms

**Figure 1:**
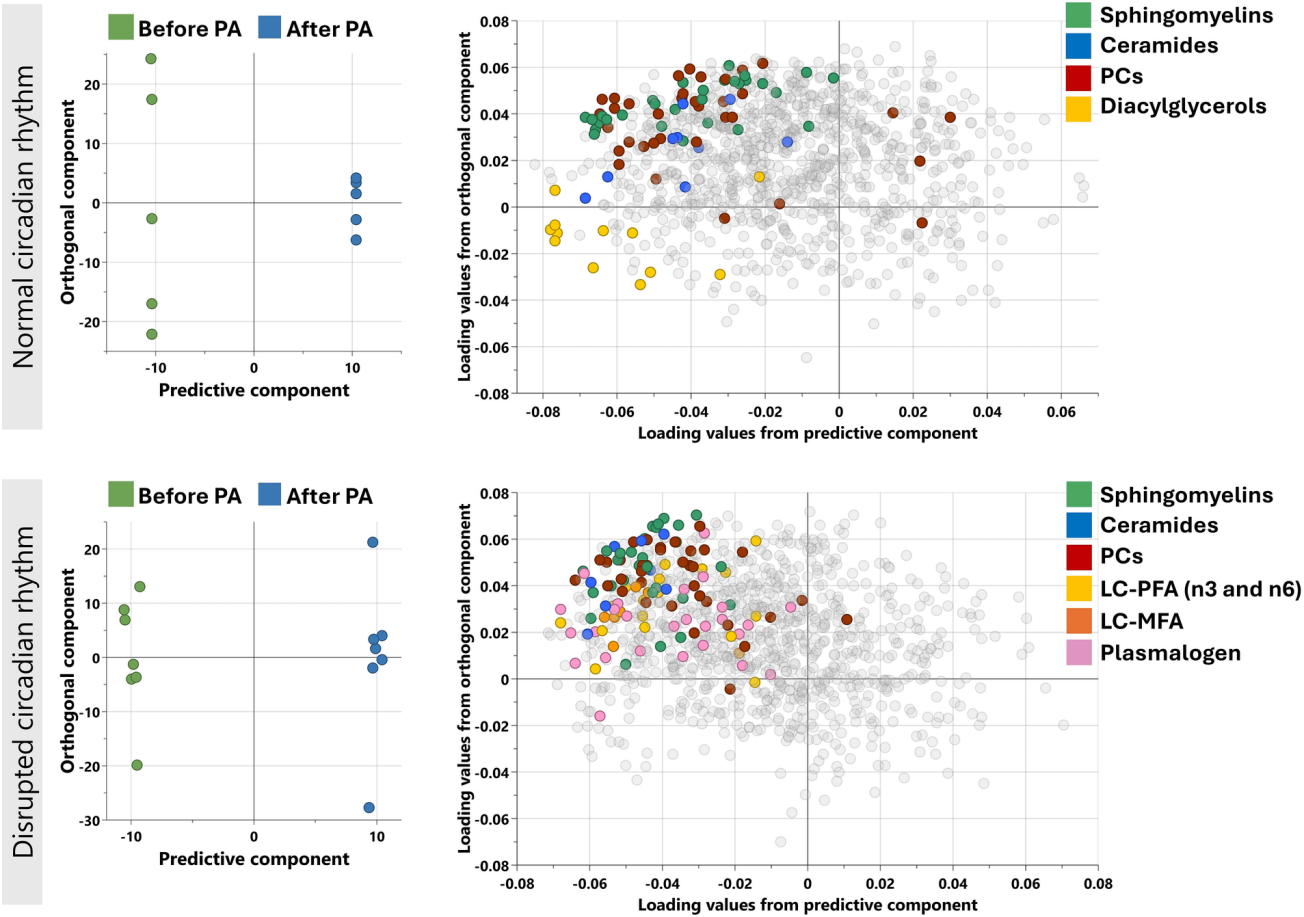
Orthogonal projections to latent structures discriminant analysis (OPLS-DA) performed for individuals with normal and disrupted circadian rhythm separately as shown. Model on normal CR individuals identified one predictive and five orthogonal components (R2X = 0.743: Q2 = 0.462) while on disrupted CR, the model identified 1 predictive and 3 orthogonal components (R2X = 0.42: Q2 = 0.564). The corresponding loadings plots (right) for normal and disrupted CR individuals as shown next to the scores plot (left). Enriched sub-pathways are colored in both OPLS-DA models, including LC-MFA, long chain monosaturated fatty acids; LC-PFA, long chain polyunsaturated fatty acid; PC, Phosphatidylcholines.

Orthogonal partial least squares-discriminant analysis (OPLS-DA) was performed separately for individuals with normal circadian rhythm and disrupted circadian rhythm to determine the change in the metabolic profile after moderate physical activity (Figure 1). The models performed in both CR groups showed Q2 > 0.4 conveying reliable reproducibility of the model.

### 3.3 Metabolites associated with physical activity (PA) in normal and disrupted CR during Ramadan

The change in all metabolites before and after PA was performed one-by-one using paired *t*-test and FC analysis to investigate metabolites associated with PA in different CR conditions. Reductions in N-linoleoyl taurine in normal CR, and lysophospholipids [1-palmitoyl-GPE (16:0); 1-docosahexaenoyl-GPE (22:6)*] and diacylglycerol [palmitoyl-linoleoyl-glycerol (16:0/18:2) (Cuesta et al., 2015)*] in disturbed CR were observed. The results are displayed in Supplementary Table S1.

### 3.4 Functional enrichment analysis

Functional enrichment analysis was performed on all nominally significant metabolites from comparative analysis in normal and disrupted CR separately. The enriched pathways with FDR < 0.01 were considered statistically significant and are shown in Table 2. Lipids such as Phosphatidylcholine, Ceramides, and Sphingomyelins are enriched during Ramadan fasting irrespective of CR condition.

**Table 2:** Enriched pathways associated with physical activity in normal and disturbed CR conditions.

Circadian rhythm	Sub-pathways	*p*-value	FDR
Normal	Sphingomyelins	3.87 × 10^−5^	1.03 × 10^−3^
	Phosphatidylcholine (PC)	1.55 × 10^−7^	1.64 × 10^−5^
	Ceramides	2.28 × 10^−6^	8.06 × 10^−5^
	Diacylglycerol	1.10 × 10^−6^	5.84 × 10^−5^
Disrupted	Sphingomyelins	9.86 × 10^−10^	1.04 × 10^−7^
	Phosphatidylcholine (PC)	1.08 × 10^−5^	5.68 × 10^−4^
	Plasmalogen	1.10 × 10^−4^	3.84 × 10^−3^
	Ceramides	3.87 × 10^−4^	8.86 × 10^−3^
	Long Chain Polyunsaturated Fatty Acid (n3 and n6)	4.22 × 10^−4^	8.86 × 10^−3^
	Long Chain Monounsaturated Fatty Acid	5.39 × 10^−5^	9.43 × 10^−3^

### 3.5 Evidence of CR related response to physical activity in Ramadan

To investigate if the metabolic alteration observed due to physical activity is dependent on sleep pattern, mixed effects model was utilized to evaluate the interaction between PA and CR in all metabolites. Twenty-three metabolites were nominally significant, however, after multiple testing correction the significance was not retained. The nominally significant metabolites are displayed in Supplementary Table S2. Similar analysis was performed in clinical parameters and significant results are displayed in Table 3. Specifically, HOMA-IR demonstrated a significant increase of 0.416 (SE = 0.192, *p* = 0.047), indicating a negative impact of disrupted sleep on insulin resistance in fasting individuals engaging in physical activity. TNF-alpha levels, while not statistically significant, showed a trend toward increased inflammation, with an estimate of 1.761 (SE = 0.867, *p* = 0.061).

**Table 3:** Effect of activity on clinical variables depending on circadian rhythm.

	Estimate (Effect size)	SE	*p*-value
HOMA-IR	0.416	0.192	0.047
TNF-alpha	1.761	0.867	0.061

## 4 Discussion

Circadian clock and quality sleep regulate important biological processes. A disruption of this clock raises the possibility of developing chronic illnesses. We have looked into Ramadan as a distinct entity in light of the increasing trend of recommending “intermittent fasting” for overall cardiometabolic health. Engaging in physical activity is also regarded as a vital habit for improved health (Warburton and Bredin, 2016). Routine physical activity is associated with a significant reduction in premature mortality risk and can help prevent over 25 chronic medical conditions (Warburton et al., 2016). Even though exercise induces beneficial changes, its positive outcomes may not be attained if the internal circadian rhythm is disrupted due to misplaced sleeping cycles (Kline, 2014).

The objective of this study was to examine the effect of disrupted circadian rhythms on physical activity during Ramadan intermittent fasting. The major findings reveal that: (i) homeostatic model assessment for insulin resistance (HOMA-IR) is improved, and decrease in inflammation marker TNF-*α* is observed in individuals adhering to normal sleeping cycles compared to their disturbed counterparts, (ii) disrupted CR individuals showed reduced plasmalogens, and long chain poly- and monosaturated fatty acids, while reduced diacylglycerols were observed in normal CR participants, and (iii) physical activity in Ramadan is associated with reduced plasma sphingomyelins, ceramides, and phosphatidylcholines regardless of circadian rhythm disruption. To our knowledge, this is the first study investigating the impact of disturbed sleep-wake cycle during Ramadan on physical activity response on a metabolomic level in general population. The interpretations of these findings are summarized below.

### 4.1 Evidence of impact of disturbed CR on insulin resistance

Our observations suggest that combined effect physical activity and normal CR has a greater reduction in HOMA-IR and an increase in disturbed CR, this is in line with studies that have demonstrated that poor sleep quality have significantly elevated HOMA-IR compared to well-rested counterparts (De Bernardi Rodrigues et al., 2016; Rawat et al., 2019). Physical activity can mitigate some negative effects of disturbed sleep; however, its effectiveness may be reduced. For example, young adults who are physically active but with poor sleep cycle may not achieve the same insulin sensitivity benefits as those who maintain both good sleep cycle and regular exercise (Rawat et al., 2019). Interestingly, TNF-*α* also shows a similar trend with higher reduction in normal CR and an increase in disrupted CR. Ramadan alone can cause reductions in TNF-α (Zeb et al., 2020), but accompanied with poor sleeping schedules shows the elevation of the inflammatory protein even in fasting state in this study. Systemic low-grade inflammation is a frequent underlying cause of obesity and the associated cardiometabolic diseases, such as, cardiovascular disease, type 2 diabetes, and non-alcoholic fatty liver disease is (Furman et al., 2019). Our data suggest that disturbed CR can contribute to obesity and metabolic disorders, even in the context of combined physical activity and fasting.

### 4.2 Lipid metabolism with respect to disturbed CR after physical activity

Findings from this study show distinct pathways that are specific to different CR conditions post physical activity. We observed a significant reduction in diacylglycerols (DAGs) in normal CR in addition to several lipid changes. Normal sleep patterns combined with exercise facilitates a reduction in DAG levels, potentially aiding metabolic health and recovery (Morton et al., 2020). Notably, circadian rhythms also govern the activity of several DAGs (Chua et al., 2013; Gooley, 2016), and as such could serve as a marker for both sleep debt and circadian time. The reduction of diacylglycerols in individuals with normal sleep patterns post-exercise may indicate a protective mechanism against metabolic disturbances (see also Supplementary Figure S1). This result is consistent with the literature that suggests that accumulation of certain DAGs in skeletal muscle exacerbates insulin resistance. This highlights the importance of both physical activity and optimal sleep cycle in maintaining healthy lipid profiles and overall metabolic health.

On the other hand, we observed reductions in plasmalogens, long chain poly-(PUFAs) and monosaturated fatty acids (MUFAs) in individuals with disturbed CR in response to PA. This relationship highlights the complex connection between lipid metabolism, sleep quality, and physical exertion. We have shown that plasmalogen levels decrease in individuals with disturbed sleep patterns. These specialized phospholipids are crucial for maintaining membrane integrity and cellular function (Bozelli et al., 2021). Lower levels of these lipids are associated with cognitive decline, as seen in aging and neurodegenerative disorders. A recent study showed that supplementation with plasmalogens can enhance cognitive performance and promote neurogenesis and synaptic plasticity in animal models (Gu et al., 2022). Plasmalogens also have anti-inflammatory responses (Bozelli et al., 2021). A reduction in these lipids, coupled with increase in TNF-*α*, seen in our study with disrupted CR individuals indicates a potential increase in inflammation due to misaligned CR (see also Supplementary Figure S2). Additionally, PUFAs and MUFAs, which are essential fatty acids, lower the production of inflammatory cytokines and eicosanoids (Calder, 2006) and are therapeutic to acute and chronic inflammatory conditions. This study has shown these lipids are also lowered in disrupted CR, which leads to inflammation and metabolic dysregulation and eventually leading to insulin resistance, obesity and overall reduction in cognitive health (Gooley, 2016).

### 4.3 Lipid metabolism during Ramadan in relation to physical activity

Univariate and pathway enrichment analysis showed that metabolites from lipid pathways, mainly, sphingomyelins, ceramides, and phosphatidylcholines are significantly lower after exercise in both normal and disrupted CR individuals, indicating a central favorable lipid metabolism and energy regulation due to fasting and physical activity. Intermittent fasting is associated with metabolic transition from glucose to fatty acid-derived ketones, where there is a change from storing fat and synthesizing lipids and cholesterol to mobilizing fat through fatty acid oxidation and fatty acid-derived ketones, which maintain muscle mass and function (Anton et al., 2018). Recent studies have shown that RIF is accompanied by unique lipidomic fingerprints that set it apart from non-fasting days due to the notable dietary and lifestyle changes that coincide with the month of Ramadan, such as changes in macronutrients intake (Shatila et al., 2021), quality of sleep and duration (Faris et al., 2020), and physical activity (Almuraikhy et al., 2024; Farooq et al., 2021). Considering the mentioned factors, our data is expected to show a list of lipidomic changes, as such, our data demonstrates reduction in ceramides and sphingomyelins, and this is consistent with the recent literature which has demonstrated RIF is associated with reduced levels of sphingolipid/ceramide in circulation (Madkour et al., 2023). An excess of free fatty acids is frequently linked to fasting, and this may inhibit acid ceramidases (Chavez et al., 2005). Ceramides trigger *β*-cell death via producing free radicals, triggering endoplasmic reticulum stress, and suppressing Akt, contributing to insulin resistance (Galadari et al., 2013). Lower levels of these lipid species may improve metabolic function by increasing blood flow to skeletal muscle (Cantalupo et al., 2017). Additionally, studies have shown that reduced ceramide levels are beneficial as it ameliorates lipotoxicity, inflammation and oxidative stress, thereby contributing to improved insulin sensitivity by enhancing glucose utilization in muscle and liver tissues (Neeland et al., 2018; Haus et al., 2009).

We acknowledge the limited sample size used in this study, which may have contributed to the loss of statistical significance after applying multiple-testing correction methods. This underscores the exploratory nature of our study and highlights the need for larger-scale investigations to validate and expand upon our preliminary findings. We also acknowledge that the study’s focus on a specific cohort of female students from Qatar University limits the generalizability of the findings.

The absence of Borg rating of perceived exertion data is another limitation, which we plan to address in future studies. It’s worth emphasizing that the fasting duration was kept uniform for all participants throughout Ramadan. This deliberate decision was made to ensure consistency in fasting times and to promote adherence among all subjects. While participants were encouraged to maintain a well-balanced diet, they were not required to follow a specific, predetermined meal plan. This lack of dietary control could be viewed as an additional limitation of our research. Despite these limitations, this pilot study aimed to explore the metabolic responses to physical activity during RIF under normal and disrupted sleep patterns to provide initial insights into an under-researched area.

## 5 Conclusion

Human body is regulated by the central and peripheral circadian clocks, allowing alertness, energy consumption, nutritional processing, and activity to take place during the biological daytime due to low melatonin levels, and during the biological night, when melatonin levels are high, sleep, low energy metabolism, and restorative activities take place. Disrupted circadian rhythms have detrimental effects on health. Our data shows exercising during fasting hours have favorable lipid utilization and thereby suggesting a possible reversal of insulin resistance and lower risk of type 2 diabetes. However, under disrupted CR, the response to PA is accompanied by systemic stress due to depletion of PUFAs, MUFAs, and plasmalogens that are integral to lipid synthesis, membrane composition, and endoplasmic reticulum homeostasis, leading to increase in inflammation, oxidative stress, and metabolic dysregulation. Future research should validate current findings with larger, diverse cohorts and comprehensive assessments of sleep (polysomnography, actigraphy, questionnaires), diet (fasting protocols, nutritional composition), and physical performance (aerobic/anaerobic capacity, strength, endurance). Follow-up studies are also warranted to investigate the persistence and long-term impact of these metabolic changes.

## Data Availability

The original contributions presented in the study are included in the article/Supplementary material, further inquiries can be directed to the corresponding author.

## Supplementary material

SUPPLEMENTARY FIGURE S1 Spearman’s correlation was performed between metabolic markers and diacylglycerols in the normal CR group post physical activity. * signifies a *p*-value <0.05.

SUPPLEMENTARY FIGURE S2 Spearman’s correlation was performed between inflammatory markers vs. plasmalogens and PUFAs in the disrupted CR group post physical activity.
