# Supplementary figures and images for: The influence of circadian rhythm disruption during Ramadan on metabolic responses to physical activity: a pilot study
Source: Front Neurosci. 2025 Feb 24;19:1542016. doi: 10.3389/fnins.2025.1542016 (PMC11891360; doi:10.3389/fnins.2025.1542016)

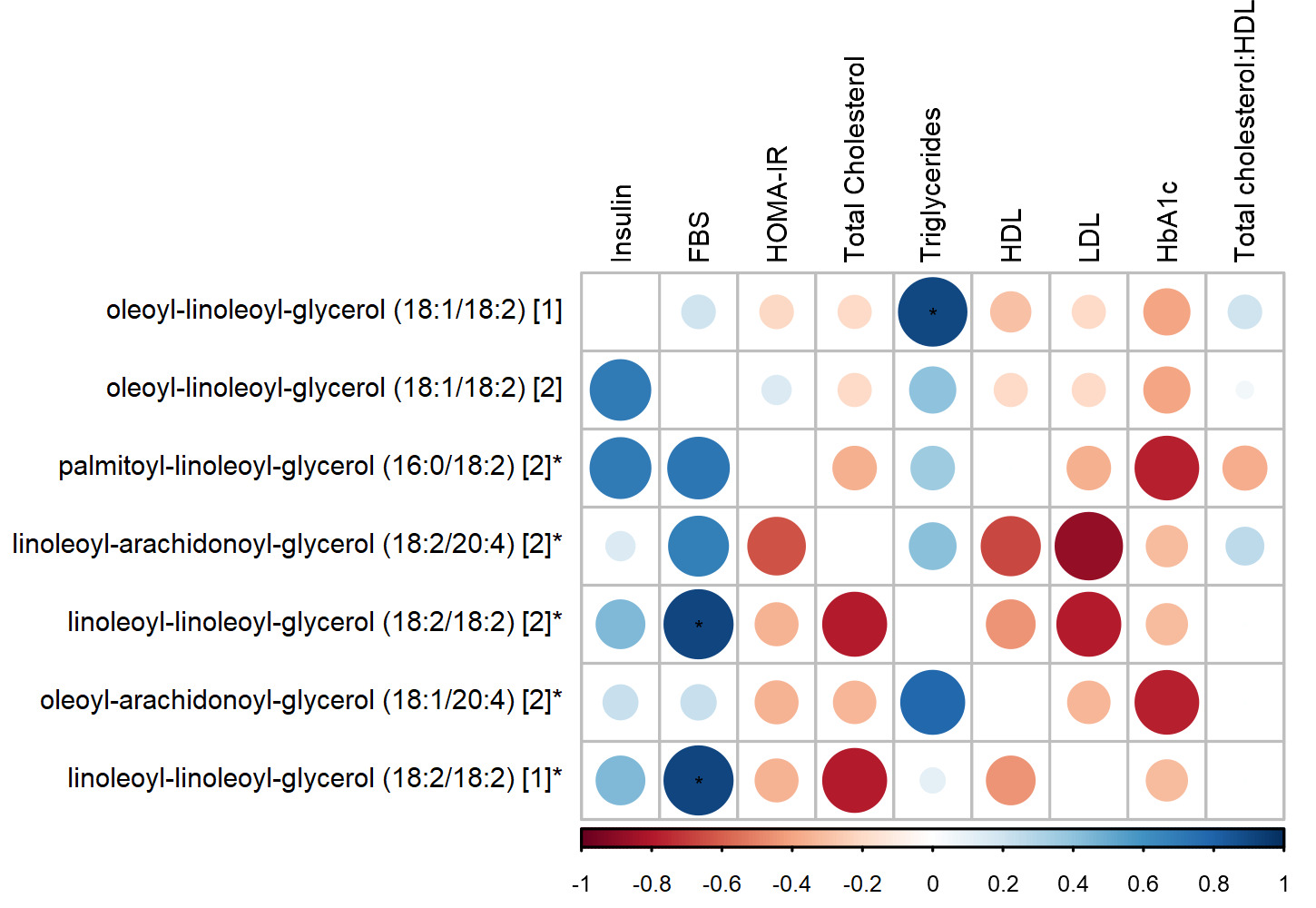

Supplement: SUPPLEMENTARY FIGURE S1 — Spearman’s correlation was performed between metabolic markers and diacylglycerols in the normal CR group post physical activity. * signifies a p-value <0.05. [file Image_1.jpeg]

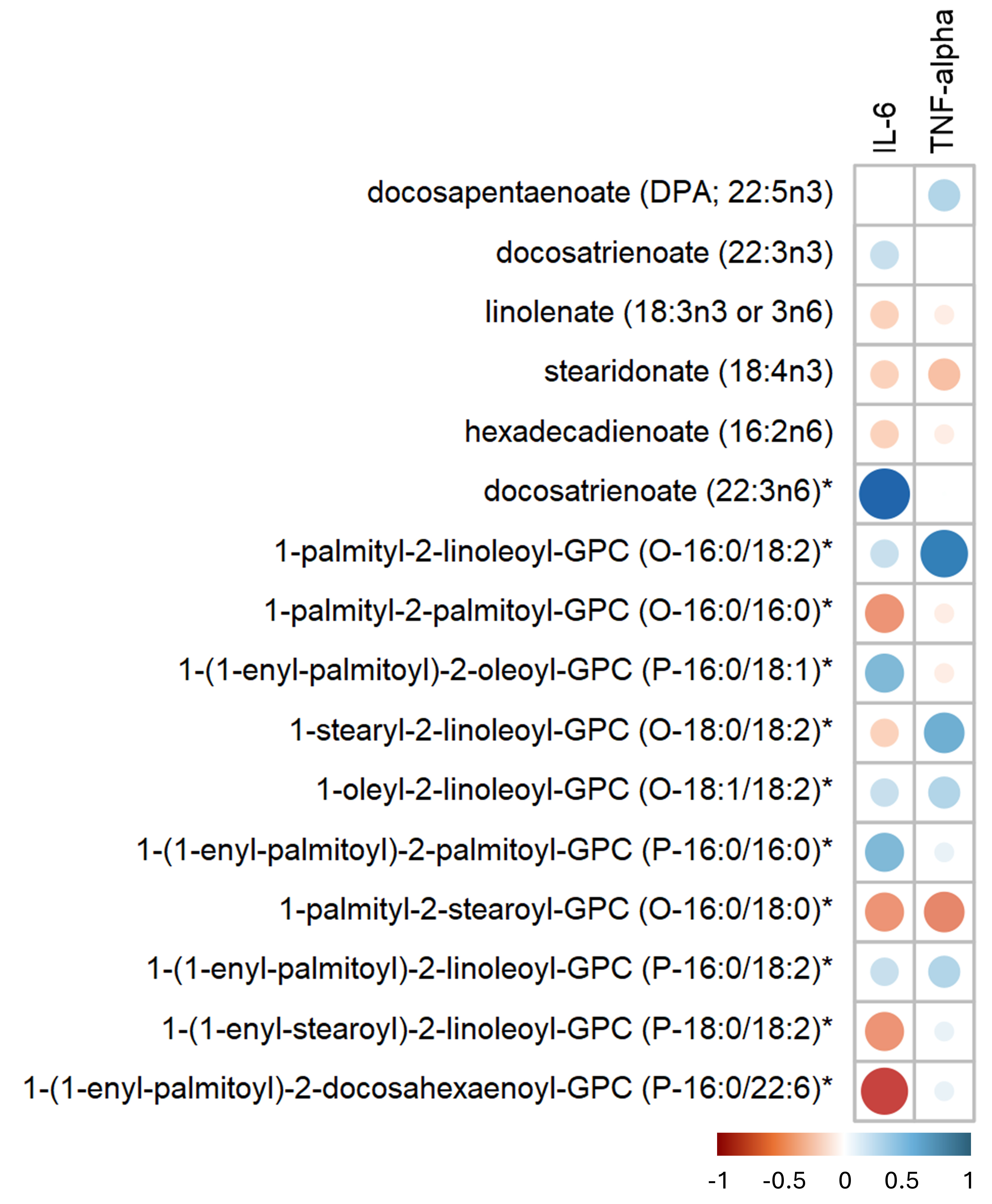

Supplement: SUPPLEMENTARY FIGURE S2 — Spearman’s correlation was performed between inflammatory markers vs. plasmalogens and PUFAs in the disrupted CR group post physical activity. [file Image_2.jpeg]
