# Supplementary material for: The influence of circadian rhythm disruption during Ramadan on metabolic responses to physical activity: a pilot study
Source: Front Neurosci. 2025 Feb 24;19:1542016. doi: 10.3389/fnins.2025.1542016 (PMC11891360; doi:10.3389/fnins.2025.1542016)
Supplement: Supplementary file 3 [file Data_Sheet_1.docx]

**Supplementary Table S1:** Top metabolites associated with physical activity in normal and disrupted CR.

| metabolite | Super-pathway | Sub-pathway | Log2FC | p-value | FDR | CR |
| --- | --- | --- | --- | --- | --- | --- |
| N-linoleoyltaurine* | Lipid | Endocannabinoid | -1.318 | 8.11E-05 | 0.082 | normal |
| 1-palmitoyl-GPE (16:0) | Lipid | Lysophospholipid | -0.587 | 2.13E-04 | 0.073 | disrupted |
| 1-docosahexaenoyl-GPE (22:6)* | Lipid | Lysophospholipid | -0.929 | 1.56E-04 | 0.073 | disrupted |
| palmitoyl-linoleoyl-glycerol (16:0/18:2) [1]* | Lipid | Diacylglycerol | -2.009 | 1.38E-04 | 0.073 | disrupted |

**Supplementary Table S2:** Nominally significant metabolites that are associate with physical activity depending on sleep pattern.

| Metabolite | Super-pathway | Sub-pathway | estimate | SE | p-value |
| --- | --- | --- | --- | --- | --- |
| N2-methylguanosine | Nucleotide | Purine Metabolism, Guanine containing | 0.594 | 0.131 | 6.24E-04 |
| N-methylproline | Amino Acid | Urea cycle; Arginine and Proline Metabolism | 1.829 | 0.509 | 2.26E-03 |
| 4-vinylphenol sulfate | Xenobiotics | Benzoate Metabolism | 1.580 | 0.445 | 2.45E-03 |
| N-linoleoyltaurine* | Lipid | Endocannabinoid | 1.091 | 0.329 | 5.14E-03 |
| Cytidine | Nucleotide | Pyrimidine Metabolism, Cytidine containing | 1.051 | 0.335 | 6.76E-03 |
| 2-arachidonoylglycerol (20:4) | Lipid | Monoacylglycerol | 1.605 | 0.553 | 8.77E-03 |
| cerotoylcarnitine (C26)* | Lipid | Fatty Acid Metabolism (Acyl Carnitine, Long Chain Saturated) | 1.135 | 0.385 | 8.94E-03 |
| ascorbic acid 3-sulfate* | Cofactors and Vitamins | Ascorbate and Aldarate Metabolism | 0.744 | 0.257 | 9.09E-03 |
| N-methylhydroxyproline** | Amino Acid | Urea cycle; Arginine and Proline Metabolism | 1.794 | 0.642 | 1.17E-02 |
| oxalate (ethanedioate) | Cofactors and Vitamins | Ascorbate and Aldarate Metabolism | 0.480 | 0.167 | 1.42E-02 |
| beta-cryptoxanthin | Cofactors and Vitamins | Vitamin A Metabolism | 0.839 | 0.313 | 1.58E-02 |
| 1-arachidonylglycerol (20:4) | Lipid | Monoacylglycerol | 0.520 | 0.205 | 1.96E-02 |
| 1-linoleoylglycerol (18:2) | Lipid | Monoacylglycerol | 0.622 | 0.242 | 2.10E-02 |
| 2,3-dihydroxy-2-methylbutyrate | Amino Acid | Leucine, Isoleucine and Valine Metabolism | 1.391 | 0.568 | 2.36E-02 |
| docosatrienoate (22:3n3) | Lipid | Long Chain Polyunsaturated Fatty Acid (n3 and n6) | -1.020 | 0.414 | 2.37E-02 |
| Threonate | Cofactors and Vitamins | Ascorbate and Aldarate Metabolism | 0.492 | 0.194 | 2.50E-02 |
| acetylcarnitine (C2) | Lipid | Fatty Acid Metabolism (Acyl Carnitine, Short Chain) | 2.128 | 0.890 | 2.83E-02 |
| 4-methylnonanoylcarnitine | Lipid | Fatty Acid Metabolism (Acyl Carnitine, Medium Chain) | -1.944 | 0.827 | 2.91E-02 |
| Arabinose | Carbohydrate | Pentose Metabolism | 1.387 | 0.600 | 3.16E-02 |
| phenol glucuronide | Amino Acid | Tyrosine Metabolism | 0.999 | 0.439 | 3.41E-02 |
| N-stearoyl-sphinganine (d18:0/18:0)* | Lipid | Dihydroceramides | -1.100 | 0.476 | 3.52E-02 |
| vanillic alcohol sulfate | Amino Acid | Tyrosine Metabolism | 1.360 | 0.613 | 3.82E-02 |
| glucuronide of C10H18O2 (8)* | Partially Characterized Molecules | Partially Characterized Molecules | 1.050 | 0.496 | 4.70E-02 |

**Supplementary Table S3:** Baseline comparison between the normal CR and disrupted CR groups. Data are presented as mean (SD)/median (IQR) and were compared using Student’s t test/Mann Whitney U test respectively. A p-value of <0.05 was considered significant.

| **Parameter** | **Normal CR** | **Disturbed CR** | **p-value** |
| --- | --- | --- | --- |
| **Height (m)** | 1.61 (1.6-1.62) | 1.59 (1.56-1.64) | 0.744 |
| **Age** | 21 (20-22) | 22 (22-25.5) | 0.456 |
| **BMI** | 28.64 (9.97) | 24.46 (3.68) | 0.413 |
| **Weight (Kg)** | 74.04 (28.14) | 60.6 (54.4-63.25) | 0.871 |
| **MET** | 1686 (591-2868) | 1097 (983-2034) | 1 |
| **X6WT Distance m** | 586.2 (75.38) | 574.71 (55.64) | 0.781 |
| **Handgrip L** | 18.76 (3.97) | 22.61 (5.38) | 0.184 |
| **Handgrip R** | 20.8 (4.15) | 22.54 (5.77) | 0.556 |
| **Insulin mU L** | 14.39 (4.19) | 12.33 (5.71) | 0.488 |
| **FBS mmol L** | 5.3 (4.8-5.3) | 5.3 (4.9-5.45) | 0.407 |
| **HOMA IR** | 3.93 (3.9-3.97) | 2.09 (1.96-3.83) | 0.144 |
| **Total Cholesterol (g/dl)** | 178 (159-191) | 164 (153.5-166.5) | 0.626 |
| **Triglycerides (g/dl)** | 66 (62-67) | 55 (48-60) | 0.417 |
| **HDL (g/dl)** | 47.2 (8.32) | 60.67 (16.91) | 0.127 |
| **LDL (g/dl)** | 112 (20.92) | 100.14 (42) | 0.536 |
| **HbA1C %** | 5.15 (0.29) | 5.02 (0.42) | 0.569 |
| **IL 6 (pg/ml)** | 15.38 (11.78) | 33.46 (11.01) | 0.066 |
| **TNF alpha (pg/ml)** | 2.19 (0.43-5.76) | 0.43 (0.43-3.07) | 0.422 |
